# Natural and Engineered Halloysite Clay Interact with Bacteria in a Double-Edged Manner

**DOI:** 10.1021/acsabm.5c02355

**Published:** 2026-03-11

**Authors:** Simona Filice, Annalisa Pinsino, Viviana Scuderi, Mauro Biondo, Salvatore Walter Papasergi, Mario Scuderi, Maria Laura Amoruso, Roberta Farina, Sebania Libertino, Silvia Scalese

**Affiliations:** † Consiglio Nazionale Delle Ricerche, Istituto per La Microelettronica e Microsistemi (CNR-IMM), Ottava Strada n.5, Catania I-95121, Italy; ‡ Consiglio Nazionale Delle Ricerche, Istituto di Farmacologia Traslazionale (CNR-IFT), c/o Area Della Ricerca di Palermo, Via Ugo La Malfa, 153, Palermo 90146, Italy; § Università Degli Studi di Messina, MIFT, Viale Ferdinando Stagno D’Alcontres, Messina 98166, Italy; ∥ Dipartimento di Scienze Chimiche, Università Degli Studi di Catania, Viale A. Doria 6, Catania 95125, Italy

**Keywords:** halloysite, aluminosilicates, clay, antimicrobial activity, bacteria, sea urchin

## Abstract

In this paper, we investigated the behavior of a natural, low-cost, and biocompatible clay, focusing on its potential use in biomedical applications, with an eye on its ability as a material that inhibits or promotes bacterial growth. The interaction of raw and acid-treated halloysite (HT) with Gram-positive and Gram-negative bacteria representative of different environments, such as the human body, food, air, soil, water, and marine environments, was explored. Environmental strains of *Escherichia coli*, *Acinetobacter baumannii*, *Lactococcus lactis*, and *Staphylococcus aureus* were isolated and examined for their responses to HT and its derivatives after acid treatment, including acidic HT (HT (H^+^)), precipitate (P), and supernatant (S). HT before and after acid treatment did not have any effect on the growth of this subset of opportunistic bacteria that mainly inhabit air and water. Bacteria of marine origin (*Vibrio* spp and *Halomonas* spp) were isolated from the body lesions of a spotted diseased sea urchin, *Paracentrotus lividus* *.* These species were highly sensitive to the material tested, showing an opposite survival response under treatment with the raw or the acidic HT forms. Materials were fully characterized by scanning and transmission electron microscopy (SEM and TEM) and X-ray photoelectron spectroscopy (XPS). The responses of marine bacteria exposed to HT and its derivatives were dependent on their structural and physicochemical properties, as elucidated here.

## Graphical abstract

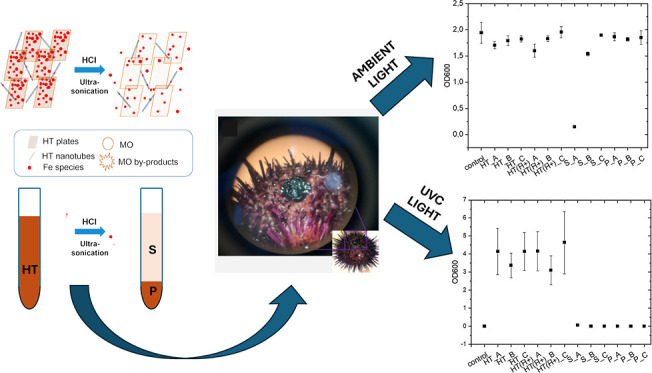

## 1 Introduction

Bacteria are organisms formed by a single cell whose dimensions are in the microscale order (∼0.4–3 μm^3^).^1^ Although their size is a million times smaller than ours, these organisms play a significant role in human survival and ecosystem health by decomposing organic matter and cycling nutrients.^1^ Host–bacteria interaction drives simultaneously the host resistance evolution and the development of pathogens’ alternative strategies, such as peaceful coexistence with the host (e.g., beneficial symbiotic association) or evading to escape host surveillance and triggering the infection (e.g., antimicrobial resistance).^2^

For example, a microbiome is a beneficial symbiotic association that can prevent infections by competitively excluding invading microorganisms by occupying specific sites (e.g., skin and gut), making it difficult for pathogens to colonize and cause disease on the host.^3–5^

On the other hand, antimicrobial resistance occurs as a natural process through genetic changes, which are then accelerated by human activities, including the overuse and misuse of antibiotics. The 2022 Global Antimicrobial Resistance and Use Surveillance System (GLASS) report estimated that antimicrobial resistance was the leading cause of 1.27 million global deaths in 2019 and contributed to 4.95 million deaths.^6,7^ Over 65% of hospital infections are caused by biofilms that invade medical devices, as reported by the Centers for Disease Control and Prevention in the United States.^8^

Thus, the need to find new and more effective ways to control the bacterial proliferation and colonization of surfaces within biofilm development is becoming a priority for the scientific community. Besides health issues, biofouling is also a problem of considerable technical and economic significance in industrial systems where filters are used.^9^ For example, the undesirable deposition of micro and macro-organisms on industrial cooling equipment results in operational failures such as clogging in water flow in the cooling water conduits and condenser tubes, increasing power consumption, reducing heat transfer efficiency, and accelerating pipeline corrosion.^9^

To address the limitations of conventional drugs in inhibiting biofilm formation in healthcare settings, agriculture, water, and food, several types of alternative materials of the new generation are being developed using advanced nanotechnologies.^10^ In particular, carbon nanomaterials such as graphene, carbon quantum dots, graphene oxide (GO), and reduced graphene oxide (rGO) are very suitable for limiting microbial infection^11–13^ and are also biocompatible. To maintain the properties of these nanomaterials, reduce the cost and add new functionalities, a plethora of polymeric nanocomposites filled with carbon and/or metal oxide nanoparticles (NPs) were generated and tested,^14–16^ or even sulfonated pentablock copolymers able to induce a combined repulsion and contact-killing mechanism against biofilm formation,^17–20^ and natural and biocompatible materials, including clays.^21^

Clays are aluminum phyllosilicates composed of planes of silica and alumina. These minerals are rich in iron, magnesium, alkali metals, alkaline earth, and other cations.^22,23^ The investigation and use of halloysite-based materials are increasing exponentially. This growth is driven by their high surface area, high adsorption capacity, chemical inertness, high cation exchange capacity, interlayer reactions, low cost, and low toxicity, which make them suitable for removing water contaminants through adsorption^22^ and/or photocatalysis,^23^ for CO_2_ capture,^24^ for food packaging,^25^ as drug delivery systems,^26^ and antimicrobial agents.^27–29^

Natural clays have been known to have both positive and negative effects on microbial vital functions since ancient times.^30^ However, the mechanisms explaining the chemical–physical nature of clay/bacteria interaction have not yet been well understood,^31^ which is a major obstacle to comparing clays of different origins. What is known is that each deposit appears mineralogically different since clays are derived from hydrothermally altered volcaniclastic environments, which is why different clays show different effects on bacteria.^32^

Clays can alter the humidity, nutrients, and pH of the microbial microhabitats,^33^ and they can also adsorb microbial metabolites and inorganic substances functional for microbial survival.^34^ By coming into contact with the bacterial surface, they can act as either a reservoir film for nutrients and water or a protective layer against harmful processes.^35,36^ On the other hand, clay has the potential to become toxic if it exceeds certain critical values, acting as a barrier to bacterial uptake of nutrients or as a reservoir of chemical elements that affect bacterial metabolism.^29,37^ Lastly, clay minerals provide structural support to protect environmental bacteria from UV irradiation,^38,39^ thanks to their cation exchangeability.

Recent archeological research evidenced the use of clays to cure wounds, soothe irritations, and clean skin by *Homo sapiens* between 320,000 and 305,000 years ago.^40^ Notably, clays have been used as anti-inflammatory agents and for cosmetic purposes since ancient Greece,^41^ but also for health benefits in all indigenous cultures worldwide.^42^ For example, necrotizing fasciitis caused by *Mycobacterium ulcerans* can be cured by a French green clay poultice.^43^ However, not all French clays highlight antibacterial properties; on the contrary, they can stimulate bacterial growth.^44,45^

The more effective antibacterial clay recently identified was extracted from an open-pit mine of hydrothermally altered pyroclastic material in the Cascade Mountains.^46^ This material acts as an antimicrobial agent against several bacterial species, including *Escherichia coli*, *Staphylococcus aureus*, *Pseudomonas aeruginosa*, and *Salmonella typhimurium* *,* within 24 h.^46^ The killing activity of this clay is related to the release of iron (Fe) ions from pyrite, which causes the production of hydroxyl radicals, whereas the bacteriostatic properties are dependent on its siloxane surface.

Since the literature does not provide clear and definitive statements on the activity of clay toward bacteria, here, we characterize the chemical–physical properties of a natural clay coming from the Dunino mine (Poland),^22,23^ here namely Halloysite (HT) clay, formed by both platy and tubular structures mainly composed of Si, Al, O, and Fe particles,^22,23^ for correlating them with its potential ability to inhibit or promote bacterial growth.

Based on the encouraging suggestion coming from the literature^46^ and taking into consideration the natural structure of this HT clay based on Fe particles, the clay has been treated with hydrochloric acid to facilitate the extraction and dissolution of Fe oxide contained in the aluminosilicate layers, and all the forms obtained after acid treatment, including acidic HT (HT(H^+^)), precipitate (P), and supernatant (S) were used for biological testing.

To thoroughly examine the bacterial interaction with HT clay (and its derivatives), a wide variety of bacteria from different environments, such as the human body, food, air, soil, water, and marine settings, were exposed to various concentrations and environmental conditions.

We demonstrate that HT clay acidification induces the leaching of Fe ions from Fe-bearing silicates within the clay structure, and the release of these ions from the mineral into the solution is responsible for bacterial growth inhibition, highlighted for the first time using bacteria of marine origin. In addition, both raw HT and HT(H^+^) dissolved in bacterial culture and exposed to UV-C irradiation a few times, help marine bacteria start growing after cold stress. Based on our findings, the HT clay nanotechnology makes them suitable for pharmaceutical use after engineering.

## 2 Experimental Section

### 2.1 Materials

The HT clay used for the present study comes from the Dunino mine (Poland)^22,23^ and stands out for its specific plate-tubular structure with the ratio of HT to kaolinite of 66:34. Dunino is one of the largest halloysite deposits in the world, located in Lower Silesia (this is one of the currently exploited deposits of this mineral in the world). It contains at least 10 million tons of homogeneous raw material and is an open-pit mine. HT clay extracted from this deposit is a product of basalt weathering.

Hydrochloric acid 37 wt % and Rhodamine B isothiocyanate were acquired at Merck Life Science S.r.l. (Milan, Italy).

Before each experiment, the HT clay powder was weighed and dispersed in a suitable water volume using ultrasonic treatment for 6 h (h) to obtain the desired clay concentration. The resulting dispersion was further ultrasonicated for 1 h before each measurement.

Different concentrations of acidified HT (here named HT(H^+^)) samples (1, 0.5, 0.25, and 0.125 mg mL^–1^) were prepared by dispersing raw HT powder in water using an ultrasonic treatment for 4 h. The pH value of 5 mL of these solutions (which had initial pH values of 5–6) was changed by adding drops of hydrochloric acid (final pH < 3). The acidified solutions were ultrasonicated for 4 h, and a precipitate was observed after one night. The precipitate and supernatant (here named P and S, respectively) in the acidified solutions were separated, and the former was dispersed in water.

### 2.2 Characterization

The physicochemical properties of samples were investigated by UV–vis absorbance spectroscopy, scanning and transmission electron microscopy (SEM and TEM, respectively), and X-ray photoelectron spectroscopy (XPS). Their antimicrobial activity was tested for both the raw (HT) and acidified forms (HT(H^+^), P, and S.

The UV–visible spectra of HT, HT(H^+^), P, and S were recorded using a UV–vis spectrophotometer (Cary 50 UV/vis by Agilent Technologies, Santa Clara, CA, USA), in a wavelength range between 200 and 800 nm, using wide optical window quartz cuvettes (200–2500 nm).

For each sample (HT, HT(H^+^), P, and S), the concentration of iron (Fe) ions dissolved in solutions versus raw clay concentrations at a fixed ultrasonication time was evaluated by comparing the 334 nm peak of the UV–visible spectra with the calibration curve obtained for FeCl_3_ solutions at different known concentrations.

A field emission SEM instrument (Zeiss Supra35 FE-SEM) was used to investigate the material morphology.

Structural and chemical analysis of each sample was performed using TEM. To prepare the specimen for TEM observation, an aqueous solution containing the clay powder at neutral and acidic pH was dropped onto a lacey-carbon TEM grid. TEM analysis was performed using a probe Cs-corrected TEM JEM-ARM200F by JEOL (Akishima, Tokyo), equipped with a cold FEG electron source operated at a primary beam energy of 200 keV in TEM and scanning TEM (STEM) mode and equipped with a 100 mm^2^ silicon drift detector for EDX spectroscopy.

The chemical composition of samples was investigated by the PHI Genesis Multi-Technique Scanning XPS system, with a monochromatic Al Kα X-ray beam and a 180° hemispherical electron energy analyzer. The system is equipped with a dual-beam charge neutralization system that allows turn-key neutralization of all types of insulating samples.

The antioxidant effect of raw HT was evaluated by recording the UV–visible spectrum of its solution (5 mL) with Rhodamine B (10^–5^ M) in the absence or presence of hydrogen peroxide (30%, 0.5 mL). The same spectra were acquired as a reference for Rhodamine B solutions without samples and recorded as already reported.

### 2.3 Biological Activity Tests

#### 2.3.1 Test on Gram-Positive and Gram-Negative Bacteria

##### 2.3.1.1 Strains and Growth Conditions

The following bacterial strains were used as bacterial models to perform the assays: *E. coli* EC1000, *Acinetobacter baumannii* ATCC17978 (ATCC17978), *A. baumannii* clinical isolated from an infusion pump in intensive therapy (AB#2), and *A. baumannii* clinical isolated from the forearm of a healthcare worker’s clothing in intensive therapy (AB#6), as representative of Gram-negative bacteria; *Lactococcus lactis* MG1363, *S. aureus* ATCC25923 and ATCC29213, and the environmental isolate *S. aureus* (SaLC), as representative of Gram-positive bacteria. A Lysogeny broth (LB) was used as the culture medium for all of the bacterial models. For any experimental setting, the chosen strains were isolated from frozen stock on LB agar and left to grow for 24 h at 37 °C (*E. coli*, *A.baumannii*, *S. aureus*) or RT (*L. lactis*).

##### 2.3.1.2 Growth Inhibition Assay

The starting cultures (SC) were prepared by inoculating a single colony in 1 mL of LB broth and letting growth reach the stationary phase at 37 °C or RT for 24 h. To verify the possible effect of the HT samples on the ability of the bacteria to grow in LB, the SC were diluted to reach the final optical density of about 0.05 (OD_600_) in LB broth with the dispersed HT, HT(H^+^), P, and S, at the concentrations of 0.100, 0.050, 0.025, and 0.0125 mg mL^–1^. Also, controls were diluted to reach the final OD_600_ of about 0.05 in LB broth. The negative controls were LB broth or LB broth mixed with the clay samples. The pH of the clay fractions dispersed in the LB broth was measured to determine the capacity of the LB medium to buffer the pH of the clay fractions. All the antimicrobial assays were performed in 96-well plates, and the turbidity (OD_600_) was measured using an Agilent BioTek plate reader (model Sinergy ht). In each experiment, any condition was triplicated, and each test was performed three times. Since HT(H^+^), P, and S dispersed in LB broth strongly affected the pH of the LB broth, these samples were not used for these assays.

##### 2.3.1.3 Biofilm Formation Assay

To verify the possible effect of HT on the bacterial capability to form a biofilm, SC were prepared by inoculating a single colony of each chosen strain in 1 mL of LB broth and letting the growth reach the stationary phase at 37 °C or RT for 24 h. The SC were diluted to reach the final optical density of about 0.05 (OD_600_) in LB broth and in LB broth with the dispersed HT, at the final concentrations of 0.100, 0.050, 0.025, and 0.0125 mg mL^–1^. The biofilm formation under the tested conditions was compared with the biofilm formation in LB. The negative controls were the LB and the LB mixed with HT at a 0.100 mg mL^–1^ concentration. The inoculi and the controls were then dispensed in triplicate in a polystyrene 96-well plate and incubated at 37 °C or RT for 48 h. The level of biofilm formation was estimated by light emission, adapting the CellTiter-Glo 2.0 Cell Viability Assay (Promega Corporation), using the following protocol. The nonadherent bacteria were discharged, wells were washed twice with PBS, and a volume of CellTiter-Glo Reagent was mixed with an equal volume of PBS. To facilitate the lysis of the adherent bacteria, plates were mixed on an orbital shaker in the dark (10 min, RT). Then, the mix was transferred to a white opaque plate, and light emission was measured by an Agilent BioTek plate reader (model Sinergy ht). In each experiment, any condition was triplicated, and each test was performed three times.

#### 2.3.2 Test on Bacteria from the Sea Urchin

##### 2.3.2.1 Sample Collection

Adult sea urchins (*Paracentrotus lividus*) were collected along the coast of Sicily (Italy) and maintained in a sea urchin invertebrate breeding facility equipped with closed-circulation aquaria supplied with flow-through oxygenated seawater and controlled temperature and salinity conditions. Spotting disease has been a major problem for sea urchins housed in breeding facilities for a long time. Bacteria associated with a body lesion on the surface of a diseased sea urchin (spotting disease) held in captivity for 9 months were collected by scraping the lesion with a sterile toothpick. The toothpick was then dipped in 5 mL of sterilized 4% LB medium (Sigma, USA) dissolved in artificial seawater (ASW) and incubated for 5 days under continuous agitation at 21 °C. Concomitantly, one region far from the degraded epidermal tissue (putative negative control) was scraped by toothpicks and dipped in LB for culturing as already described. A tube with only LB was also used as an absolute negative control. After 5 days of culturing, all samples were stretched on agar plates prepared by diluting 3.5% LB-agar in ASW until colonies appeared to be visible. One part of the culture was diluted in LB in ASW medium and stored at 20 °C in tubes supplemented by 20% sterile glycerol in ASW (frozen stock).

The sea urchin is a marine invertebrate; therefore, it does not raise ethical issues. No regulation or formal ethical approval is necessary for experimentation. We applied the practical and vigilant 3R (replacement, reduction, and refinement) principle, limiting the use of living organisms and improving animal welfare.

##### 2.3.2.2 Characterization of Bacteria Isolated from the Sea Urchin Lesion

Single colonies grew on a 3.5% LB-agar plate were selected based on the morphology, collected from the plate by a sterile tip, and prepared for Sanger sequencing of the 16S rRNA gene as the following procedure: typed colonies were put in tubes of 0.2 mL containing 50 μl of ultrapure distilled water-DNase/RNase free (Invitrogen, USA) and then microlysed for 7 min (min) at 100 °C. The same was done with a bacterial pellet collected from 1 mL of LB medium culture after centrifugation at 5000*g* for 5 min. Samples were amplified for the 16S gene using two sets of primers, sequenced, and the sequence analyzed for homology by a free Basic Local Alignment Search Tool^47^ (BACT16S-500 analysis, BMR Genomics).

##### 2.3.2.3 Exposure of the Bacteria Isolated from the Sea Urchin on LB Medium and LB-Agar Medium by HT and its Derivatives

Stock solutions (HT, (HT(H^+^)), P, and S) prepared in Milli-Q water with a concentration of 1 mg mL^–1^ were vortexed for 1 min to achieve improved particle dispersion and then dispersed in 0.8 mL LB medium at increasing concentrations (0.001, 0.01, and 0.1 mg mL^–1^). The pH of the clay fractions dispersed in LB broth in ASW was measured to determine the capacity of the LB medium to buffer the low pH. 10 μL of the bacterial suspension stored at −20 °C was added to 5 mL of sterilized 4% LB medium in ASW and left to grow for 24 h at 21 °C. After growing, aliquots of the bacterial suspension were then exposed to HT, (HT(H^+^)), P, and S and cultured for 24 h at 21 °C under visible light by shaking.

Another subset of bacteria from frozen stock was directly cultured in an LB medium (with or without testing materials) and preliminarily exposed to UV (253.7 nm UV-C) for 6 min under shaking. The optical density of each sample (600 nm wavelength) was measured by a Biophotometer (Biophotometer D30, Eppendorf, Hamburg, Germany). Assays were performed in triplicate and repeated three times. Concurrently, at time zero of exposure, 10 μL of each bacterial suspension of frozen stock was loaded on agar plates, kept under the hood, and allowed to dry. After drying, 10 μL of each particle concentration was loaded upon each spot of bacteria, kept under the hood, and allowed to dry, and the bacteria were left under exposure at room temperature for days.

Data were examined using GraphPad Prism Software 9 (La Jolla, CA, USA) and reported as the mean ± standard deviation (SD). The statistical significance of differences between data sets (*P*-value ±0.05) was estimated using ordinary one-way ANOVA, followed by Dunnett’s multiple comparison test.

##### 2.3.2.4 Colony Density Evaluation of Bacterial Culture Exposed to Iron-Oxide Nanoparticles

To investigate the possible role of Fe oxide released from HT during acidification, we performed experiments using commercial Fe oxide nanoparticles (Fe_2_O_3_ NPs) as reference.^48^ Five serial dilutions (1:10) of the original bacterial culture were made by adding 100 to 900 μL of LB in ASW seven times. Each bacterial culture dilution was vortexed, and a drop of 10 μL each was loaded on LB agar plates, kept under the hood, and allowed to dry. Fe_2_O_3_ NP stock solutions prepared in Milli-Q water were vortexed for 1 min to achieve improved particle dispersion and then dispersed in 0.8 mL LB medium at increasing concentrations (0.001, 0.01, and 0.1 mg mL^–1^). After drying, 10 μL of each particle concentration was loaded upon each spot of bacteria, kept under the hood, and allowed to dry, and the bacteria were left under exposure at room temperature for days. The colony growth of the fifth serial dilution was visualized under a microscope. The experiment was conducted three times to ensure reproducibility.

## 3 Results and Discussion

### 3.1 HT Characterization

#### 3.1.1 Acid Treatment of Raw Dunino Halloysite

The mechanisms behind the protective or killing activity of clays interacting with bacteria have not been well elucidated, as already reported. Anyway, data from literature suggest that the antibacterial activity of clays should be ascribed to the release of Fe and aluminum ions.^27–29^ Taking cues from these data, we treated HT clay with hydrochloric acid (HCl) to favor the extraction and dissolution of Fe oxide contained in this aluminosilicate clay and the selective etching of alumina layers.^22,23^ Since our scope was to extract and release only Fe ion species, leaving the clay structure (i.e., surface area and ion exchange capacity) almost unaltered, we optimized the HCl concentration and the process time to achieve our goal, as reported in the experimental section (paragraph 2.1). Indeed, it is known that higher concentrations of HCl and longer reaction times lead to the complete dissolution of all structures.^49^

To confirm this assumption, we also performed the same treatment by adding HCl until a white, insoluble precipitate was formed. SEM images of this sample are reported in Figure S1 of the Supporting Information. We observed that the remaining structures were formed by irregular plates, suggesting that the initial structures were etched. Indeed, EDX spectra acquired on these plates and reported in Table S1 confirmed the results, highlighting that the structures that were observed were silica plates, and alumina was no longer present because of the complete etching caused by the acid.

Raw HT dispersed in water was prepared at different concentrations (1, 0.5, 0.25, and 0.125 mg mL^–1^), sonicated, and partially treated with HCl. The resulting precipitate (P) and supernatant (S) were separated and redispersed in water.

The UV–visible spectra of raw HT, P, and S fractions are reported in Figure 1.

**Figure 1:**
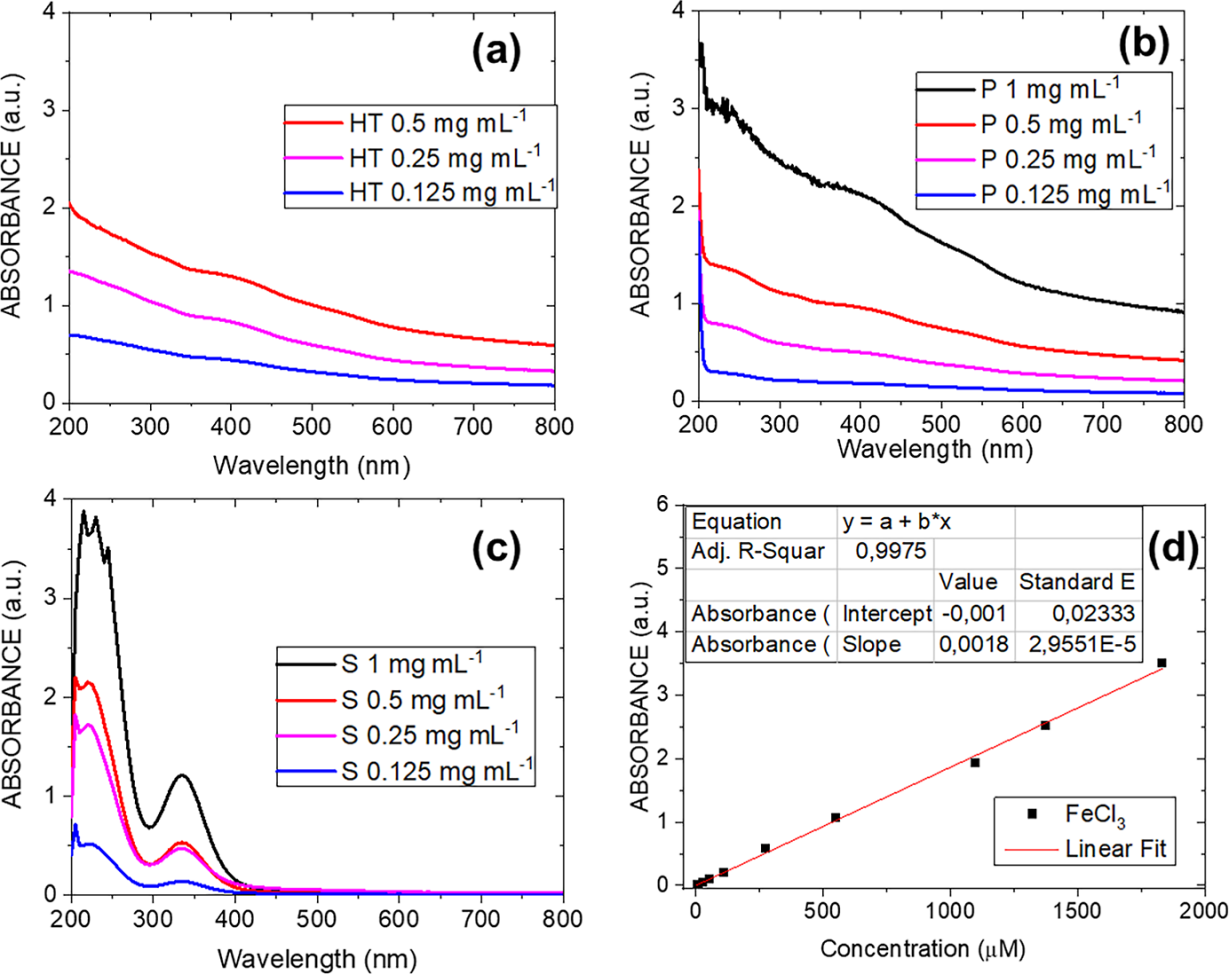
UV–vis absorbance spectra of the HT materials at different initial concentrations, before and after acid treatment: (a) raw HT, (b) precipitates (P); (c) supernatants (S); (d) a calibration curve created for different concentrations of FeCl_3_ acid solutions.

Figure 1a shows the spectra, which depict the sum of extinction contributions caused by both aluminosilicate layers and Fe oxide NPs, demonstrating how HT can absorb light in the entire range of investigation. Notably, the spectrum of HT at 1 mg mL^–1^ is not reported, since it was out on the saturating absorbance scale. The absorption features in P (see Figure 1b) were identical to those in the initial HT samples, but S’s spectra (see Figure 1c) indicated an increase in absorption in the region below 250 nm, and also an absorption peak at 334 nm, the latter being associated with Fe ions released into the solution after the acid treatment.

The Fe ion concentration for each sample was determined by comparing its absorbance peaks with those of the calibration curve shown in Figure 1d, and the results are reported in Table 1.

**Table 1:** Concentration of Fe Ions Extracted by HT Samples at Different Initial Concentrations

HT solutions (mg mL^–1^)	extracted iron concentrations (mg mL^–1^)	extracted iron concentrations (μM)
0.125	0.009	160
0.25	0.027	480
0.5	0.030	540
1	0.068	1220

Assuming a specific HCl concentration and reaction time, the amount of extracted Fe ions increased with the initial clay concentration, beginning at the surface and continuing with the Fe species present inside (between the layers of) the HT structure. The release of Fe species (mainly ions) in the solution occurred during the etching of alumina layers, as reported in previous work.^23^

These processes are described by eqs 1 and 2

1 $${Fe}_{2}O_{3}+6HCl\to {2FeCl}_{3}+{3H}_{2}O$$

2 $${3Al}_{2}O_{3}{2SiO}_{2}+18HCl\to {6AlCl}_{3}+{2SiO}_{2}+{9H}_{2}O$$

#### 3.1.2 Morphological, Structural, and Chemical Characterization

To deeply investigate the nature of Fe species in HT samples before and after acid treatment, TEM and STEM characterization combined with EDX spectroscopy were performed.

TEM analysis was carried out on the raw HT, P, and S. The former two samples showed very similar structures, and therefore, we have reported only one of them (Figure 2) for a clearer discussion. In the supernatant, it was impossible to detect significant structures due to the etching process that occurred during the acid treatment, releasing Fe species into solution (mainly in the ion form).

**Figure 2:**
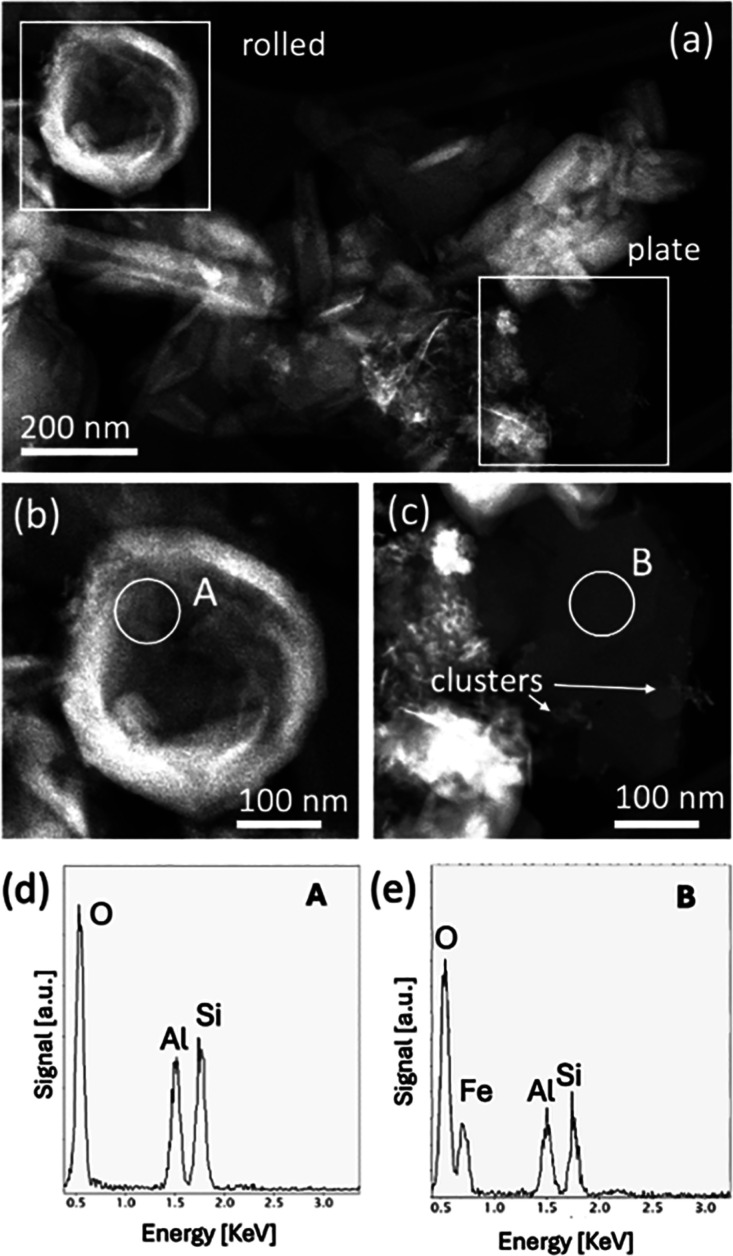
(a) STEM dark field micrograph of rolled and plate halloysite. (b) and (c) Higher magnification STEM images of the rolled and plate structures, along with the corresponding EDX spectra (d,e).

According to our previous morphological characterizations,^22,23^ HT was found to be composed of rolled and flat structures typical of the material found in the Dunino deposit. Both structures are indicated in the STEM micrograph in Figure 2a and the high magnification of the same figure (b, c, respectively).

These structures consisted of tetrahedral Si_2_O_5_ layers and octahedral Al layers. Chemical characterization obtained through EDX spectra, acquired during the STEM analysis, showed that both structures were composed of Si, Al, and O, with an Al/Si ratio close to 1 (see the EDX spectra in Figure 2d,e). The crystallographic nature of the raw HT sample was previously investigated by electron diffraction acquired during TEM analysis and reported in our previous work.^22^ Two different halloysite crystals were observed with different interlayer distances (i.e., 10 and 7 Å, respectively). Furthermore, the estimated *b*-axis of the cell resulted in an increased value of 9.25 Å, far from the tabulated 8.9 Å value reported for the pure material.^22^ This large difference in the *b* parameter could be ascribed to the presence of Fe atoms within the crystal and, in particular, to the partial isomorphous substitution of Fe^3+^ for Al^3+^ in the octahedral sheet.^22^

In the analyzed sample, it was possible to observe some clusters of particles mainly composed of heavier elements, as recognizable by the difference in mass contrast in the STEM image. These structures were distinct in the planar HT depicted in Figure 2c. EDX analysis of these clusters (not shown here) revealed the presence of O and Fe. The electron diffraction pattern acquired on these particles during TEM analysis has identified them as hematite nanocrystals. Indeed, Figure 3 shows the fast Fourier transform (FFT) of the TEM image in the inset, taken on these clusters, with grains on the order of 10 nm. The diffraction pattern corresponded to the hematite structure.

**Figure 3:**
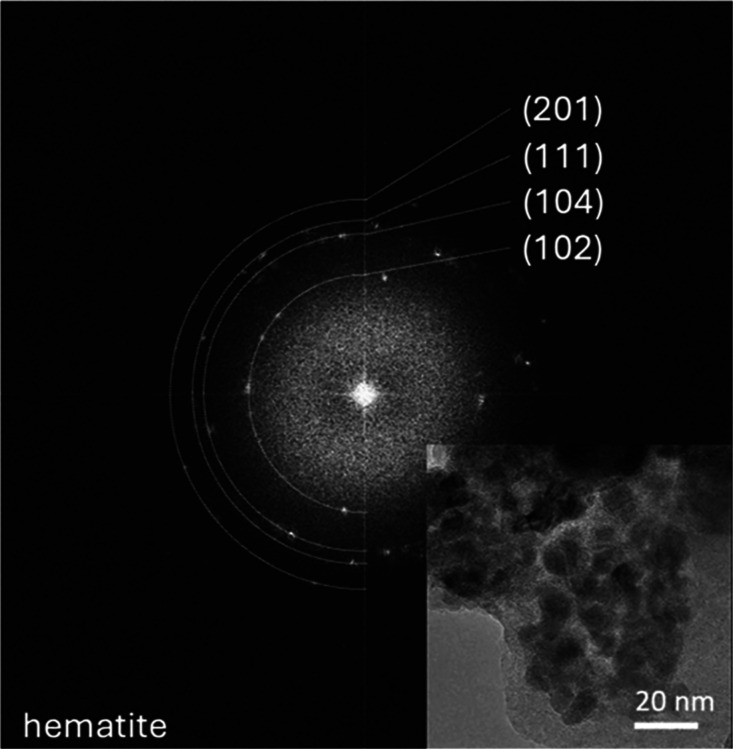
Fast Fourier Transform (FFT) of the TEM image shown in the inset, obtained on a cluster of iron oxide nanoparticles and similar to those indicated in Figure 2c.

Hematite crystallizes in a hexagonal structure with Fe atoms positioned at the center and surrounded by six oxygen atoms. In this cationic arrangement, pairs of FeO_6_ octahedra share edges with three adjacent octahedra in the same plane and one face with an octahedron in an adjacent plane along the (0 0 1) direction. In addition to the hematite particles, the Fe in allophane clay is present as Fe^3+^, substituting for Al^3+^ in the layers.

While no relevant information was obtained by TEM analysis performed on the S fraction due to the main presence of ions and the absence of relevant structures, TEM images and electron diffraction patterns acquired on the P fraction highlighted the same structural features as for the raw material (not shown here). The main differences between HT and HT(H^+^) samples before and after acid treatment are dependent on their chemical composition, as demonstrated in our previous works^22,23^ by EDX analysis and X-ray fluorescence spectroscopy. We previously demonstrated that (i) untreated HT presents a Si/Al ratio close to 1, with Fe randomly distributed; (ii) P shows a reduced amount of Fe and Al, confirming the etching mechanism here proposed in eqs 1 and 2.

In this work, we delved even further into the investigation of the surface chemical composition of HT, P, and S using X-ray photoelectron spectroscopy, focusing mainly on the external layers of our materials, which have the most impact on surface processes and environmental interactions.

XPS survey spectra for raw HT clay, P, and S samples are reported in Figure S2 of the Supporting Information.

Samples were mainly composed of silicon (Si 2p at 98 eV), aluminum (Al 2p at 74 eV), oxygen (O 1s at 530 eV), and iron (Fe 2p at 707 eV). In the S sample spectrum (blue line), additional peaks related to chlorine (Cl 2p at 199 eV), magnesium (Mg 2p at 50 eV, Mg 2s at 89 eV, Mg KL2, 3L2/3 between 301 and 347 eV), and zinc (Zn LMM between 472 and 495 eV and Zn 2p between 1022 and 1045 eV) were observed (see figure S2). These peaks are due to the chemical treatment with hydrochloric acid, which induces the dissolution of salts present in the clay.^22,23^

Table 2 provides the Al/Si and Fe/Si values for samples before (raw HT) and after acid treatment (S and P) based on the ratio of relative peak areas in XPS spectra.

**Table 2:** Al/Si and Fe/Si Values Calculated by the Ratio of the Relative Peak Area in XPS Spectra

	raw HT	*S*	*P*
Al/Si	0.17	0.22	0.13
Fe/Si	0.53	1.96	--

After acid treatment, all superficial Fe was dissolved into the solution: the Fe/Si ratio increased for the supernatant, and Fe was not quantifiable on the surface of the precipitate. Furthermore, the Al/Si ratio decreased for the precipitate, confirming that Fe dissolution occurred within aluminum etching.

This was in agreement with chemical analysis conducted on the bulk of raw HT and P samples by XRF in our previous works:^22,23^ after acid treatment, the relative Al/Si ratio decreased from 0.88 of the raw sample up to 0.39 for the P sample, as a consequence of the alumina layer dissolution. The aluminum content in the precipitate was reduced by about 50% of its initial value. Simultaneously, the P sample was depleted in Fe (about 50%) with respect to the raw samples. By XPS analysis, we also determined that in the P sample, Fe was almost completely removed from the surface. Figure 4 shows XPS spectra for Si 2p, Al 2p, O 1s, and Fe 2p_3/2_ peaks for all of the samples with the relative deconvolutions that allow for separation and evaluation of different contributions for each peak. The relative % amounts of each species are reported in Table 3.

**Figure 4:**
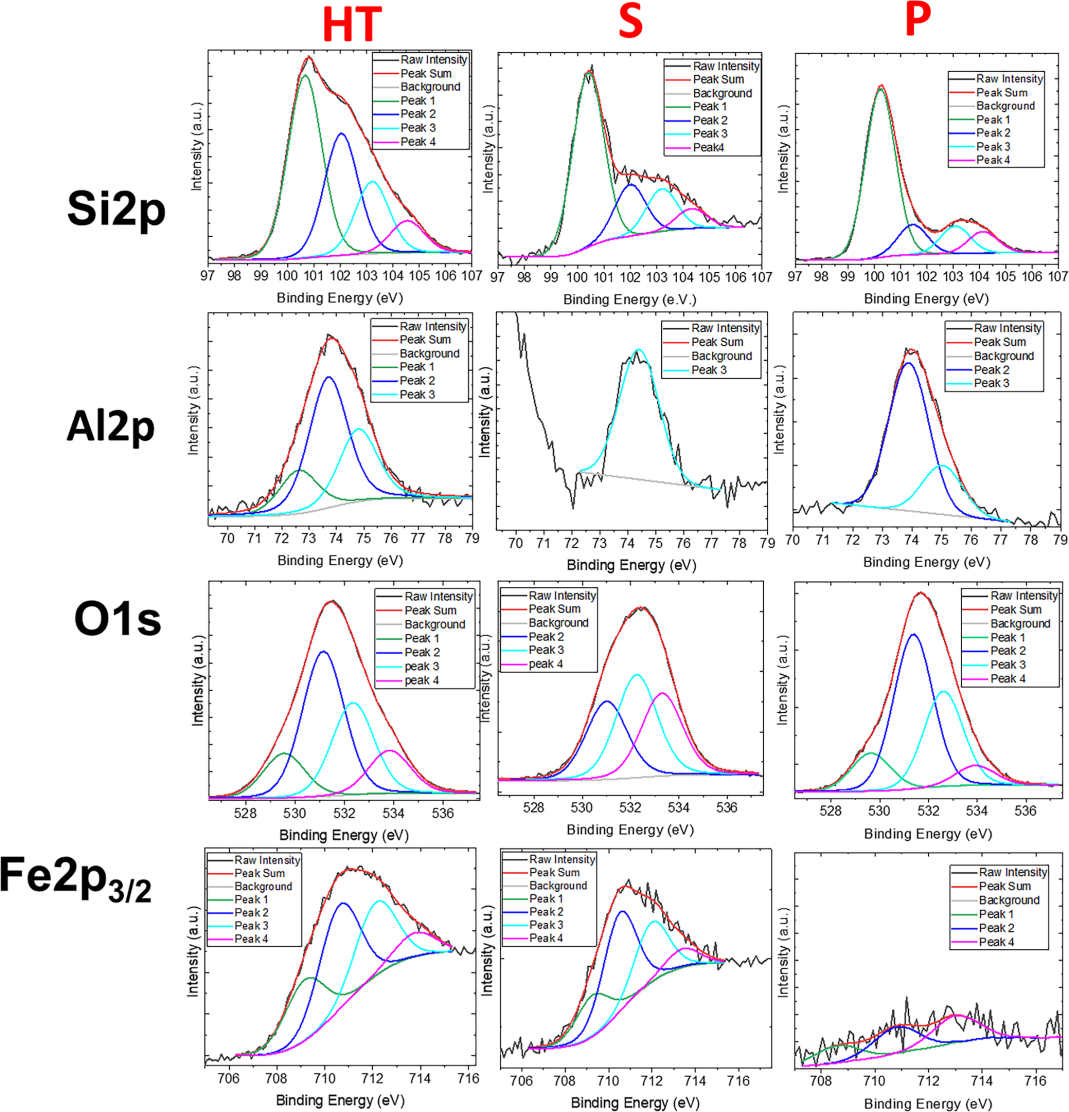
XPS spectra of Si 2p, Al 2p, O 1s, and Fe 2p 3/2 for raw HT and the supernatant (*S*) and precipitate (*P*) obtained after the acid treatment. Black continuous lines indicate the acquired spectra, red lines are the fits of the spectra, gray lines are the subtracted baseline, and the other lines are the deconvolution of the peaks.

**Table 3:** Relative Amounts (%) of Each Species Calculated by XPS Peak Deconvolution Reported in Figure 4

XPS peak	species	peak *n*# in figure	raw HT (%)	*S* (%)	*P* (%)
Si 2p	SiOH or Si^+^	1 (100.7 eV)	44.7	60.5	68.1
	SiO_2_ and SiO_4−x_	2 (102.0 eV)	29.8	17.8	12.1
	SiO_4–*x*_SiO_2_	3 (103.2 eV)	17.6	14.8	11.1
	Si^4+^ or SiO_4_	4 (104.5 eV)	7.9	6.9	8.7
Al 2p	Al metal	1 (72.7 eV)	17.6	--	--
	Tetrahedral Al/Al_2_O_3_ or oxides	2 (73.7 eV)	53.0	--	75.2
	Octahedral Al/AlCl_3_ or hydroxydes	3 (74.8 eV)	29.4	100	24.8
O 1s	Metal (Fe) oxide	1 (529.5 eV)	13.8	12.4	0
	Al_2_O_3_	2 (531.1 eV)	44.6	50.2	29.6
	SiO_2_	3 (532.3 eV)	28.3	31.0	39
	Si–O–H	4 (533.8 eV)	13.3	6.4	31.4
Fe 2p_3/2_	Fe^2+^ FeO	1 (709.2 eV)	23.2	19.0	--
	Octahedral Fe^3+^ (Fe_2_O_3_) or sub-stoichiometric oxide	2 (710.6 eV)	39.7	45.0	--
	Fe^3+^ (FeOOH)	3 (712.1 eV)	28.0	27.4	--
	Tetrahedral Fe^3+^ (Fe_2_O_3_)	4 (713.8 eV)	9.1	8.6	--

Silicon on the raw HT surface was present as Si^+^ or Si–OH at 100.7 eV, Si–O–C/Si–O–Si at 102.0 eV, as silicate (SiO_4–*x*_) and silica (SiO_2_) at 103.2 eV, and as Si^4+^ or SiO_4_ at 104.5 eV, in agreement with what was already reported in the literature.^50–53^ These peaks were also present on the surface of the S and the P after acid treatment, and we observed an increase in the first peak (Si^+^, Si–OH) at the expense of the higher silicon oxidation state species.

Aluminum was present on the raw HT surface as metallic at 72.7 eV, tetrahedral (i.e., aluminum oxides), and octahedral (i.e., aluminum hydroxide or chloride) configurations at 73.7 and 74.8 eV, respectively, in agreement with the current literature.^54^ After the acid treatment, metallic aluminum was dissolved. In the S solution, the signal of aluminum was low and present as AlCl_3_ (octahedral configuration with a peak at 74.4 eV), in agreement with eq 2 (Section 2.3.1). On the precipitate surface, we observed both tetrahedral and octahedral aluminum, with the former in a higher amount since the Al_tetrahedral_/Al_octahedral_ ratio increased from 1.8 for the raw sample to 3.0 for the P fraction.

As it is already known, oxygen is present on the surface of HT clay as Fe oxide (hematite) at 529.5 eV,^55^ as alumina at 531.1 eV,^54^ and as silica or Si–OH at 532.3 and 533.8.^50–53^

Herein, the estimated amount in hematite structures was 13.8%, with the amounts of the O–Si and O–Al bonds being almost equal (41.6 and 44.6%, respectively, with the O–Al/O–Si = 1.07). Acid treatment induced two main effects: (i) all of the hematite was dissolved and released into the S fraction, as confirmed by its absence in the spectra of the P fraction, and (ii) alumina layers were etched, as confirmed by the decrease in the amount of O–Al bonds on the surface in comparison to the amount of O–Si bonds (29.6% and 70.4%, respectively, with O–Al/O–Si = 0.42) in the P fraction. Similarly, the S fraction was enriched in hematite (12.4%, which is very close to the amount estimated on the HT surface), alumina, and Si–O species. Notably, the amount of Al–O bonds in the S fraction was higher than that in Si–O bonds, confirming that alumina was etched (50.2 and 37.4, respectively, O–Al/Si–O = 1.34). The structure of the hematite was confirmed by XPS spectra of Fe 2p_3/2_ (see Figure 4). Even if the study of Fe NPs is one of the most researched areas in recent times due to their potential applications, the interpretation of XPS spectra of these particles is still under debate.^56^ Fe species are present as oxygenated species (i.e., oxides), substoichiometric oxides, and hydroxides^56^ that are related to Fe^2+^ with multiplets at the 708.2 and 709.1 eV positions and present higher binding energy peaks related to Fe^3+^(from 710 to 714 eV). Herein, the XPS spectra of HT samples before and after acid treatment showed 4 main peaks related to both Fe^2+^ and Fe^3+^ species (see Table 3).

As shown in Figure 4, the Fe 2p_3/2_ spectrum of HT clay had both Fe^2+^ and Fe^3+^ contributions with a relative Fe^2+^/Fe^3+^ ratio of 0.302. Fe^2+^ contribution was at 709.2 eV related to FeO species, while Fe^3+^ contribution was present as three main species: Fe_2_O_3_ or substoichiometric Fe_2_O_3_ at 710.6 eV, FeOOH at 712.1 eV, and Fe_2_O_3_ at 713.8 eV.^57^ After the acid treatment, the Fe species were dissolved in the solution. Indeed, as shown in Figure 4 for the P fraction, the signal was too low and noisy for a quantitative deconvolution, confirming that all the hematite was dissolved and released from the clay surface to the solution and, therefore, can be found in the S fraction. The residual Fe species on the raw HT clay was both Fe^2+^ and Fe^3+^ with a relative Fe^2+^/Fe^3+^ ratio of 0.316. On the S fraction, the relative Fe^2+^/Fe^3+^ ratio was of 0.235. This result is also in agreement with the observed peak at 334 nm in the UV–visible spectrum of the S fraction for the Fe species (Figure 1).

An investigation of porous structures previously conducted on raw and acid-treated samples^58^ used a BET method to analyze specific surface area (SSA) and porosity, based on two ranges of pore diameters, including 0.5–2 nm (micropores) and 2–40 nm (mesopores). Results show that the SSA of raw HT is equal to 32.2 m^2^/g, while acid treatment induces an increase in both SSA (from 32.2 to 51 m^2^/g) and porosity.

### 3.2 Biological Experiments

#### 3.2.1 Results on Gram-Positive and Gram-Negative Bacteria Proliferation and Effects of HT Suspension on Biofilm Formation

The antiproliferative and antibiofilm formation activities were only assessed for raw HT because, by adding acidified fractions to LB broth, pH was found to be drastically reduced, making it impossible to determine if a putative effect on bacterial proliferation could be attributed to the activity of the material or to a change in the pH value.

To test the scale of antibacterial activity of HT, representative Gram-positive (*L. lactis* and *S. aureus*) and Gram-negative (*E. coli* and *A. baumannii*) models were examined, comparing their ability to proliferate in a liquid medium with or without the presence of raw HT (0.100, 0.050, 0.025, and 0.0125 mg mL^–1^ concentrations). *L. lactis* and *E. coli* were selected as Gram-positive and Gram-negative nonvirulent models, respectively, while *S. aureus* and *A. baumannii* were chosen as highly virulent and highly resistant models to antimicrobial agents.^59,60^ The level of medium turbidity, indicative of bacterial proliferation, was estimated by measuring the OD_600,_ and each condition was compared with the proliferation of the controls. None of the bacteria tested showed a significant susceptibility to raw HT, as no significant growth inhibition was found at any of the concentrations used (not shown). We only noticed a weak increase in the growth level of the *A. baumannii* AB#2 strain at the HT concentrations of 0.1 and 0.05 mg mL^–1^, but it was not statistically significant compared to controls (not shown).

As HT suspension showed no direct antiproliferative activity against planktonic forms of the bacterial models used, we inspected a possible HT activity against biofilm formation using a microplate assay under static conditions versus *A. baumannii* and *S. aureus*. The ability to form biofilm by these bacteria is a well-described virulence trait,^59^ and several kinds of clays are reported to interfere with biofilm formation.^60^ Results are shown in Figure S3 of the Supporting Information

The quantification of *A. baumannii* biofilm showed that HT had no inhibitory effect on biofilm formation, but, on the contrary, we noted a trend of the AB#2 strain and ATCC17978 strain to increase their ability to form biofilm aggregate as a function of the increased concentration of HT; these differences were not significant in comparison with the control. No apparent effects were observed at any concentration of the HT used to test the biofilm formation of the model strains of *S. aureus*.

#### 3.2.2 Results on Bacteria from Sea Urchin

##### 3.2.2.1 Identifying Marine Bacteria Isolated from the Body Lesion of the Sea Urchin *P. lividus*

Bacteria were isolated from a body lesion (see Figure 5) of a diseased sea urchin, which was supposed to be the result of an infection by opportunistic bacteria due to the weakening of the immune system.^61^ The lesion on the lateral surface was characterized by a darkened necrotic region on the body wall, consequential to the loss of spines and appendages (Figure 5a). Herein, we collected and identified the pathogenic bacteria that infected the sea urchin and grew in LB medium dissolved in ASW. Bacterial identity was determined using Sanger sequencing of the 16S gene, known to be a gold rule for distinguishing bacteria at the species level. Sanger sequencing highlighted the coexistence of two species of bacteria in culture (LB medium), *Vibrio spp.* and *Halomonas spp.* *,* both Gram-negatives (Figure 5b). *V. spp.* belongs to the family *Vibrionacee*, phylum *Proteobacteria*, one of the major groups within the phyla characterizing the sea urchin microbial community, harboring in the sea urchin coelomic fluid (human blood equivalent).^62^ Some *V. spp.* form rod-shaped colonies, including the species here identified (Figure 5b), and require signaling molecules to communicate between them or with other species for conducting collective behaviors, such as biofilm formation and infection, by suppressing the natural immunity of the host species,^63^ including humans.

**Figure 5:**
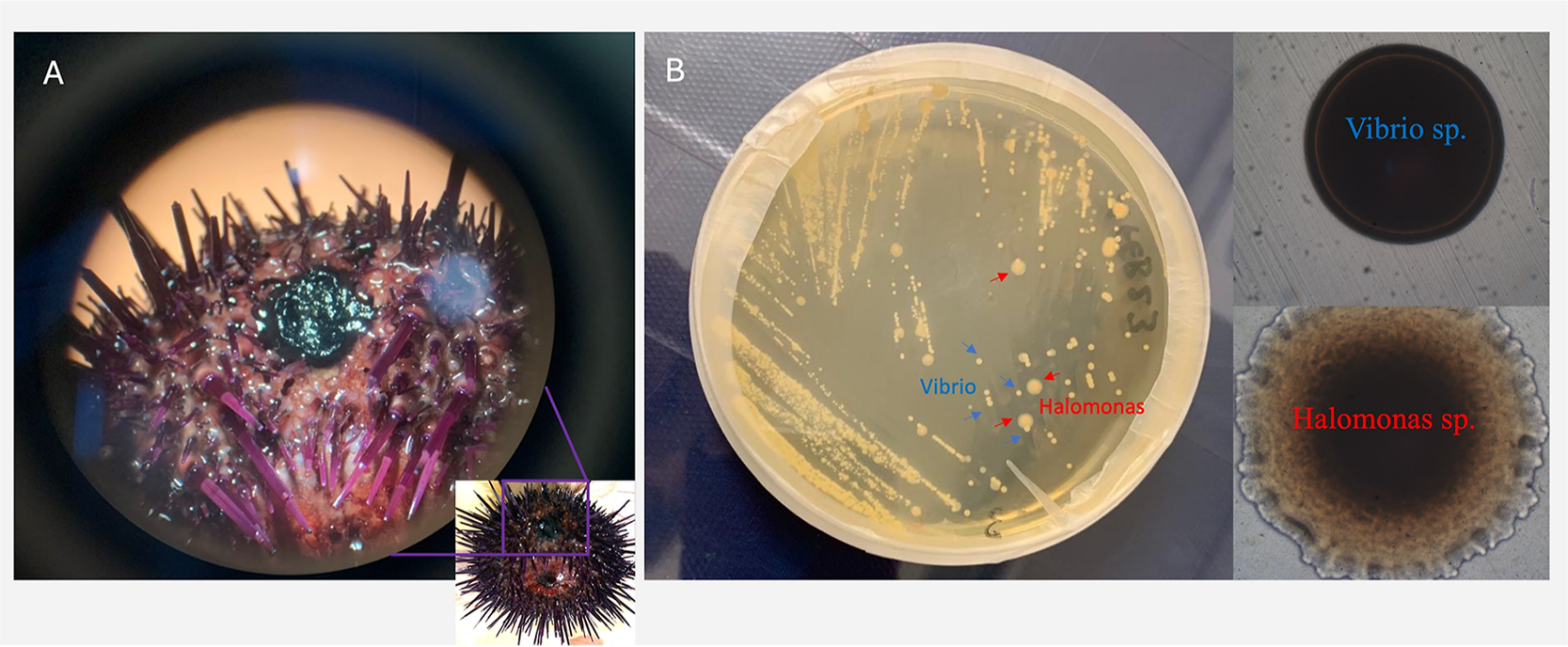
Representative images of the lesion on the sea urchin body wall and bacterial colonies. (A) Darkened necrotic tissue in a spotted diseased sea urchin. (B) Bacterial colonies in the dish of LB agar (on the left), and morphology of the cocultured colonies under the microscope (on the right).

*H. spp.* belong to the family *Halomonadaceae* *,* phylum *Proteobacteria*, a minority within the phyla comprising harboring halophilic microorganisms very different phenotypically but sharing some features (e.g., chemoorganotrophic, aerobic or facultatively anaerobic, catalase-positive and oxidase-variable, halophilic or halotolerant).^64^ Some *H. spp.* possess a strong nitrogen metabolism,^65^ driving the nitrate reduction below the influence of photoelectrons and are now considered an attractive nonconventional model for microbial cell factory engineering due to their peculiar metabolism and fast-growing capability under extreme environmental conditions (e.g., salt and pH).^66^

Fe is an essential element for regulating metabolic function in *V. spp.*; however, Fe recruitment is complicated for these bacteria due to the propensity of forming insoluble ferric complexes in nature.^67^ On the contrary, *H. spp.* (e.g., *Halomonas Titanicae* in this study) can oxidize zerovalent Fe, driving the production of Fe (II), sulfur, and amorphous Fe sulfide.^68^ Our findings suggested a symbiotic relationship between the two bacterial species related to the Fe requirement, where *V. spp* resulted predominantly (see Figure 5B). Based on the piece of evidence on the relationship of these bacteria with the Fe assumption and production, we aimed to investigate if and how the raw HT influenced the isolated *Vibrio* and *H. spp* in a coculture.

##### 3.2.2.2 Effects of HT and its Derivatives after Acidification on the Marine Bacteria Coculture

To go deep inside into the role of Fe-oxide particles present in the natural clay, the impact of raw HT in the bulk form, in the acidified form HT(H+), and the relative material derived after acidification, including S and P, was investigated in the *Vibrio-Halomonas* coculture derived from the body lesion of a spotting diseased sea urchin donor (see Figure 6). Results from *Vibrio-Halomonas* coculture exposed to raw HT, HT (H+), P, and S increasing concentrations from 0.001 to 0.1 mg mL^–1^ for 24 h showed no effects on the bacterial concentration estimated based on the measurement of the optical density of each sample at the 600 nm wavelength (OD600), except for the S with concentrations of 0.01 and 0.1 mg mL^–1^ (Figure 6). In this last case, the bacterial concentration was significantly lower than that of the controls (unexposed bacteria). Based on the capability of the LB medium dissolved in ASW to compensate for the low pH levels of the S and the P stock solutions in water (1 mg mL^–1^), we assumed that the effects of the S on the *Vibrio-Halomonas* coculture were related to the presence of Fe species present in the solution. In agreement, the colony density evaluation of the *Vibrio-Halomonas* coculture exposed to Fe-oxide nanoparticles at concentrations ranging from 0.001 to 0.1 mg mL^–1^ confirmed the previous results on the effects of the S fraction, which is enriched in Fe and Fe-oxide particles. The bacteria grown on the agar plate exposed to Fe-oxide nanoparticles are shown in Figure 7.

**Figure 6:**
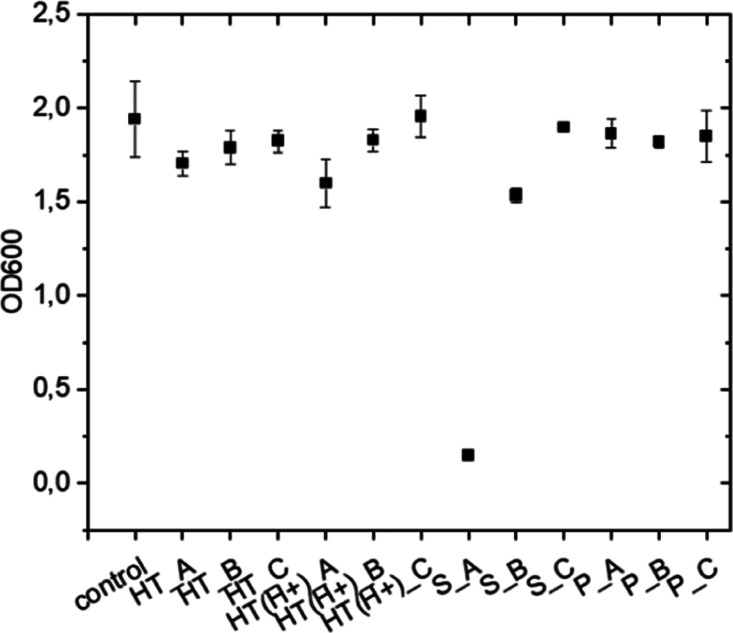
Bacterial growth in LB medium dissolved in ASW with and without HT clays and derivated at different concentrations (i.e., 0.1, 0.01, and 0.001 mg mL^–1^ here named _A,_B, and _C, respectively). The representative graph shows the OD600 bacterial cell number from one experiment reported as mean ± SD of three replicates.

**Figure 7:**
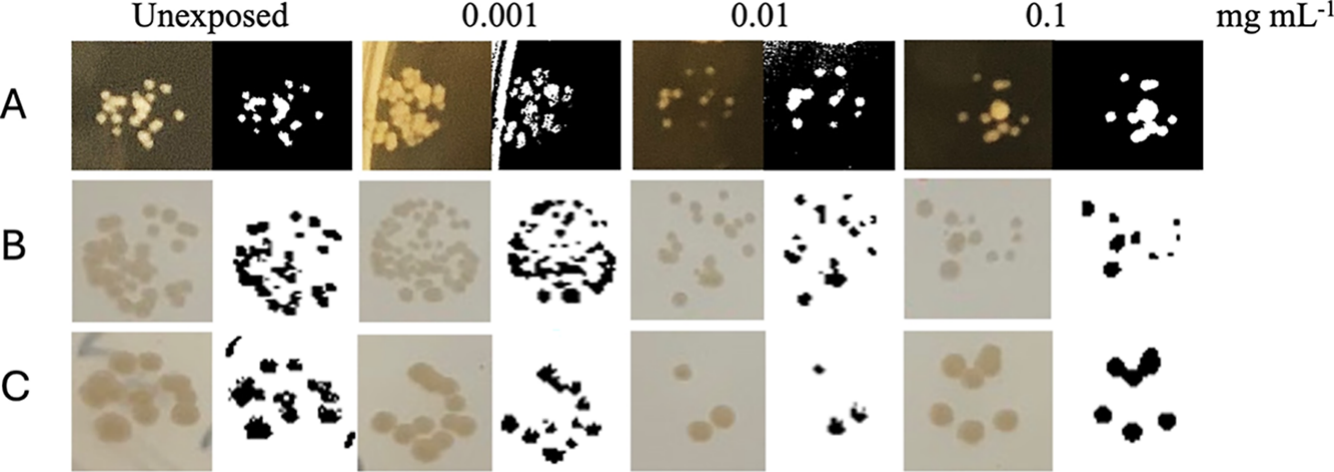
Bacterial growth on an agar plate exposed to commercial Fe-oxide nanoparticles at increasing concentrations (0.001, 0.01, and 0.1 mg mL^–1^). A, B, and C are three replicates that were performed at separate times. Capture time: A: 2 day exposure; B: 4 day exposure; C: 6 day exposure. For each sample: on the left, the original images; on the right, images with spotting colonies on agar plates in black and white to highlight colony abundance.

The interaction between clay minerals and microorganisms played a crucial role in the chemical evolution and origins of life in the Proterozoic eon (2500–540 million years ago), based on their capability to concentrate and bind compounds and protect organic molecules from UV.^35^ Consequently, they lead to microbial growth and biosynthesis by concentrating nutrients and protecting against adverse environmental conditions (e.g., UV irradiation, low pH, and temperature changes). Based on the pieces of evidence of the protective role of clays on bacteria under unfavorable physicochemical conditions, the effects of HT, HT(H+), S, and P samples were evaluated in the *Vibrio-Halomonas* just tawed (skipping the growth phase) after UV-C exposure of the LB medium containing compounds dissolved in LB for 6 min. Results from *Vibrio-Halomonas* coculture exposed to irradiated HT, HT(H+), S, and P solutions at increasing concentrations from 0.001 to 0.1 mg mL^–1^ for 24 h highlighted the assumed effects (i.e., protective role) reported in the literature for both HT and HT(H+), independent of the concentration used (Figure 8). Results were confirmed for both LB culture (Figure 8, on the left) and LB-agar culture (Figure 8, on the right). Furthermore, we hypothesize that the different response of the P fraction with respect to HT is a consequence of the alumina layers etching and the release of Fe species, the latter playing a role in the photodegradation process under UV-C irradiation. This is confirmed by previous results on the photodegradation of an anionic dye, methyl orange,^23^ by HT and acid derivatives.

**Figure 8:**
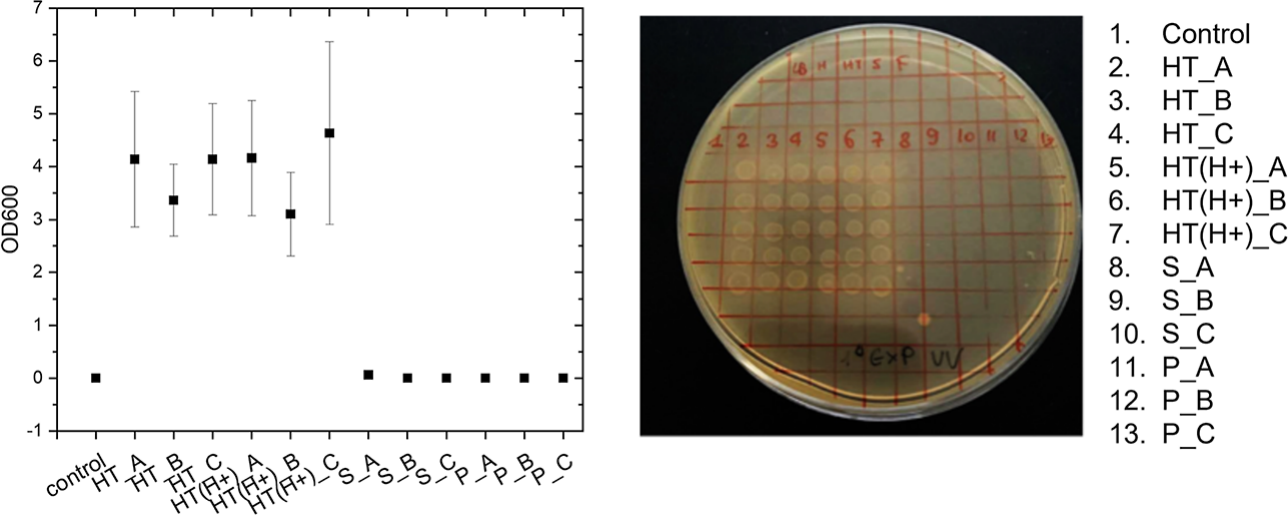
Bacterial growth in an LB medium, preliminarily exposed to UV-C (with and without HT clays and derived at different concentrations, i.e., 0.1, 0.01, and 0.001 mg L^–1^ here named _A,_B, and _ C, respectively). Representative graph showing the OD600 bacterial cell number from one experiment reported as mean ± SD of three replicates on the left, and bacteria growing on an agar plate on the right. Capture time: 7 days of exposure.

HT behavior could be ascribed to two possible reasons:

(i) HT absorbs UV light like a filter, hindering the formation of active degrading species.
(ii) Oxidant active species are generated under irradiation, but then are trapped by HT structures, preventing contaminant degradation.

Concerning the first hypothesis, HT showed a large light absorption also in the UV region of the spectrum, as reported in Figure 1, although no antibacterial effect was reported. Starting from the assumption that reactive oxidant species are formed under UV irradiation, we investigated the second hypothesis as follows: Rhodamine B, a dye used as an OH probe,^69^ was exposed to hydrogen peroxide alone and in the presence of HT.

Figure 9 reports the UV–visible absorbance spectra of Rhodamine B solutions in the presence or absence of HT clay and hydrogen peroxide.

**Figure 9:**
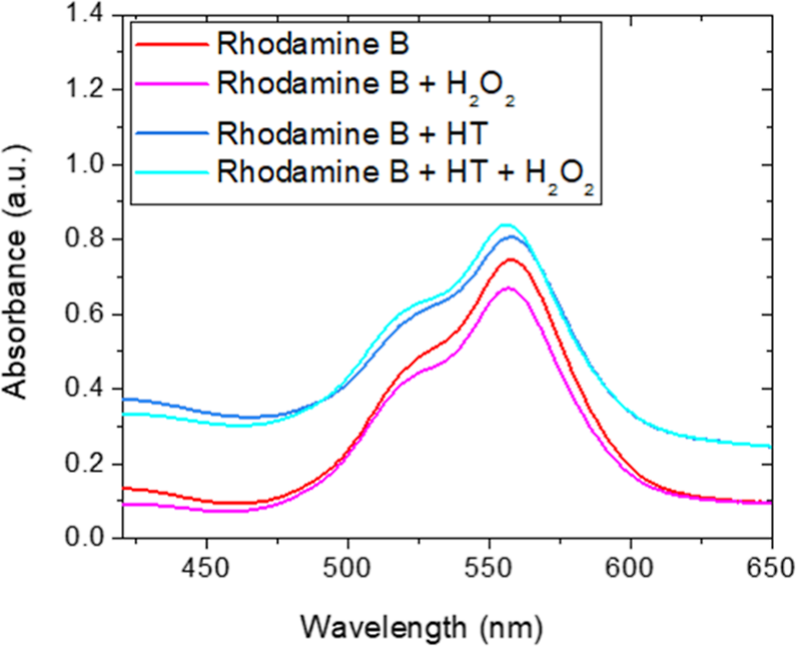
UV–visible absorbance spectra of Rhodamine B and Rhodamine + HT solutions in the presence (magenta and cyan curves, respectively) or absence (red and blue curves, respectively) of hydrogen peroxide.

Rhodamine itself is sensitive to hydrogen peroxide:^69^ the interaction with OH induces dye degradation, as shown by the reduction of its absorption peak at 557 nm (red and magenta curves, respectively). When the clay is present, no reduction was observed after adding hydrogen peroxide (blue and cyano curves, respectively), indicating that HT inhibits the interaction of hydroxyls with Rhodamine, thus preventing its degradation. This explains the protective behavior shown by untreated HT toward the *Vibrio-Halomonas* cocultured just tawed (skipping the growth phase) after UV-C exposure of the LB medium containing HT (see Figure 8). Although the hydroxyl radicals were formed during UV-C light irradiation, they can be adsorbed by the HT itself, preventing bacterial death. This result supports the assumption that clay minerals play an important role for microorganisms, protecting them from harmful environmental conditions, including UV irradiation, extreme pH values, etc.^35^ In agreement, various clays protect incorporated organic molecules against UV radiation in the range of 250–400 nm, and this ability is linked to the presence of bulk Fe_2_O_3_ absorbing and blocking the UV transmission,^70^ even if the photoprotection mechanism has not yet been thoroughly elucidated.

## 4 Conclusions

HT clay can be modified by a low-cost and simple process, allowing for the obtaining of two opposite behaviors in terms of antimicrobial activity. Their physical and chemical characteristics open the possibility of using this material to induce bacterial growth or to protect them or organic molecules (as bioplastics or drugs).

Both properties can be useful for applications in pharmacology and animal breeding (e.g., fish farms), with the double purpose of (i) reducing infections, if used instead of conventional antibiotics as highly recommended by the EMA, or (ii) enhancing the healthy state of animals in captivity, if administered as probiotics. Finding natural, low-cost, biocompatible, and viable alternatives to conventional antibiotics indeed is crucial, and it is a relevant challenge for the future. More research will be necessary to fully assess their long-term safety and understand their mechanisms of action on organisms.

## Supplementary Material



## Notes

SEM image (a) of halloysite powder treated with an excess of HCl and deposited on a polymeric substrate for EDX analysis (b), the red and green squares indicate the sample areas where EDX spectra were acquired, wt % of elements acquired by EDX spectra on two different areas of the deposit, XPS spectra for the initial clay and the precipitate and supernatant after acidification, additional peaks present in the supernatant solution labeled, and biofilm formation tests using a microplate assay in static condition versus *A. baumannii* (on the top) and *S. aureus* (on the bottom) for different dilutions of the HT suspension in LB broth and let to grow for 48 h (PDF)
