# Supplementary material for: Natural and Engineered Halloysite Clay Interact with Bacteria in a Double-Edged Manner
Source: ACS Appl Bio Mater. 2026 Mar 11;9(7):3336–48. doi: 10.1021/acsabm.5c02355 (PMC13060737; doi:10.1021/acsabm.5c02355)
Supplement: Supplementary file 1 [file mt5c02355_si_001.pdf]

Supporting Information

Natural and engineered halloysite clay interact with bacteria in a double-edged manner

Simona Filice [a], Annalisa Pinsino[b], Viviana Scuderi[a], Mauro Biondo[b], Salvatore Walter Papasergi[b], Mario Scuderi[a], Maria Laura Amoruso[a,c], Roberta Farina[a,d], Sebania Libertino[a] and Silvia Scalese[a*]*

[a] *Consiglio Nazionale delle Ricerche, Istituto per la Microelettronica e Microsistemi (CNR-IMM), Ottava Strada n.5, I-95121, Catania, Italy*

[b] *Consiglio Nazionale delle Ricerche, Istituto di Farmacologia Traslazionale (CNR-IFT), c/o Area della Ricerca di Palermo, via Ugo La Malfa, 153 - 90146 Palermo (PA) -, Italy*

[c] *Università degli Studi di Messina, MIFT, Viale Ferdinando Stagno D'Alcontres, Messina, 98166 Italy*

[d] *Dipartimento di Scienze Chimiche, Università Degli Studi di Catania, Viale A. Doria 6, 95125 Catania, Italy*

*Correspondence: silvia.scalese@imm.cnr.it

Corresponding Authors:

Simona Filice simona.filice@cnr.it
Silvia Scalese: silvia.scalese@imm.cnr.it

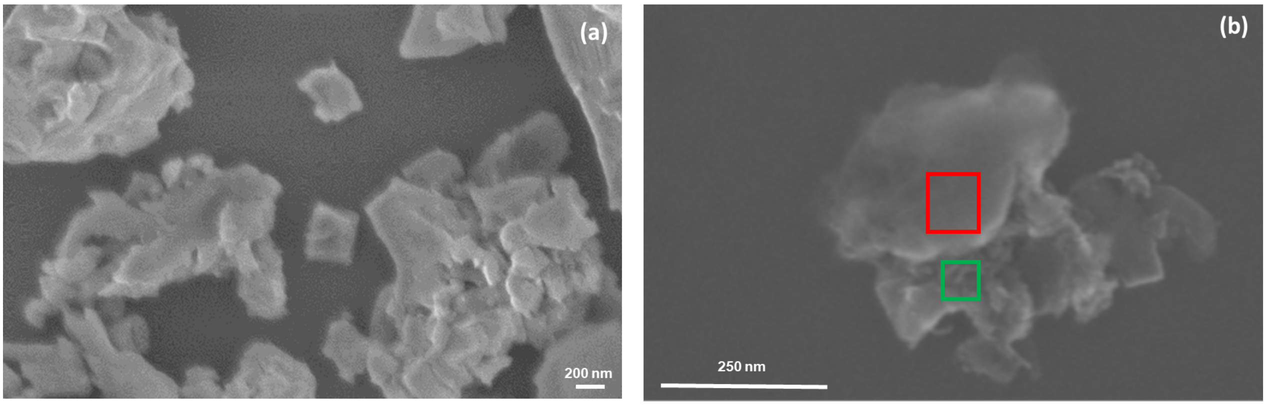

Figure S1. SEM image (a) of halloysite powder treated with hydrochloric acid (final pH < 3) and deposited on a polymeric substrate for EDX analysis (b). The red and green squares indicate the sample areas where EDX spectra were acquired.

Table S1. wt.% of elements acquired by EDX spectra on two different area of the deposit.

Element	wt% (red area)	wt% (green area)
O	6.90	3.10
Al	0.47	0.19
Si	91.89	96.03
Fe	0.74	0.69

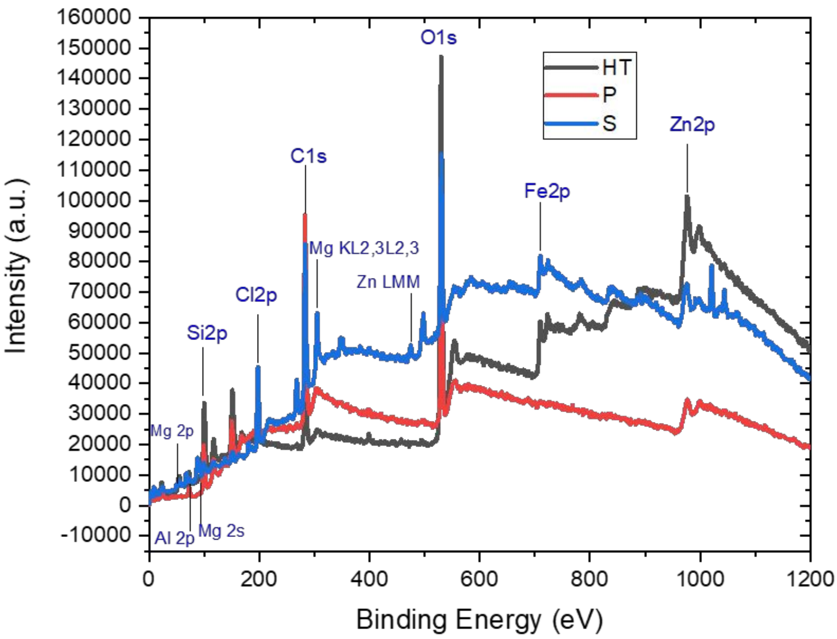

Figure S2: XPS spectra for the initial clay and the precipitate and supernatant after acidifications. Additional peaks present in the supernatant solution are labeled.

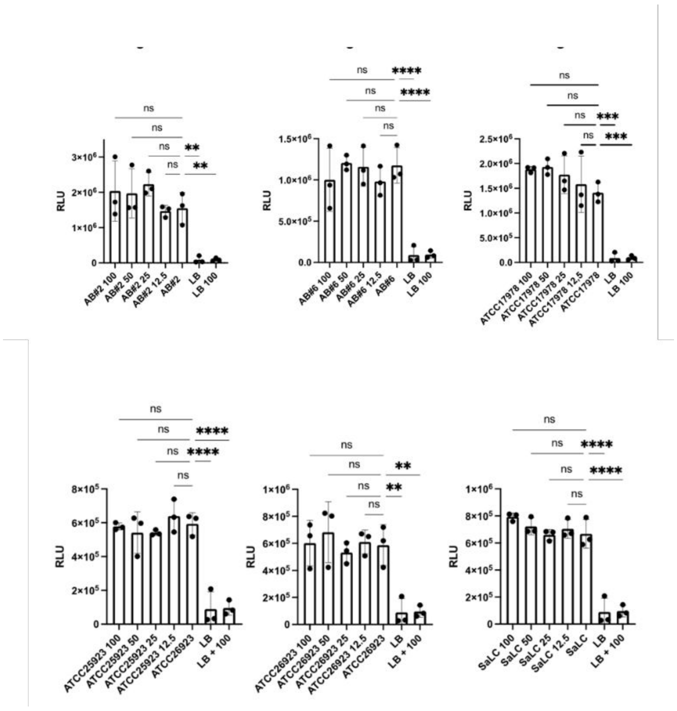

Figure S3: Biofilm formation tests using a microplate assay in static condition versus A. baumannii (on the top) and S. aureus (on the bottom) for different dilution of the HT suspension in LB broth and let to grow for 48 hours.
